# Large Language Models in Radiologist–Patient Communication: A Narrative Review for Clinical Practice

**DOI:** 10.7759/cureus.101890

**Published:** 2026-01-20

**Authors:** Jatin Naidu, Hitesh Muthyala, Sonia S Naidu, Sandeep Muralidharan, Vasanth K Baskaradoss

**Affiliations:** 1 Radiology, Division of Surgery and Interventional Science, University College London, London, GBR; 2 Radiology, Lister Hospital, East and North Hertforshire NHS Trust, Stevanage, GBR; 3 Radiology, University College London Medical School, London, GBR; 4 Obstetrics and Gynaecology, University College London Medical School, London, GBR; 5 Computer Science, University of Hull, Hull, GBR; 6 Radiology, Kettering General Hospital NHS Foundation Trust, Kettering, GBR

**Keywords:** artificial intelligence, governance, large language models, patient communication, radiology

## Abstract

Large language models (LLMs) are used in radiology to simplify reports, translate findings, and support patient-facing communication, yet their clinical value and safety remain uncertain. This narrative review was conducted in accordance with the Scale for the Assessment of Narrative Review Articles (SANRA) quality criteria and synthesises evidence from 49 studies published between 2020 and 2025, focusing on clinician-mediated use of LLMs across four domains: report simplification, multilingual translation, patient education, and patient attitudes. Across studies, LLMs consistently improved readability by 2-6 grade levels, but only one randomised trial directly assessed patient comprehension. A professional review was required in up to 80% of outputs in controlled settings, compared with <10% in observational studies. Harmful factual errors were uncommon but non-negligible (0-10% depending on task and model). Translation performance was highest for high-resource languages, while semantic drift was more frequent in low-resource languages, necessitating bilingual review. Patients generally accepted AI-assisted communication when clinician oversight was explicit. Current regulatory and professional guidance support supervised, institution-hosted deployment. Evidence supports specific use cases, patient summaries, translation drafts, and educational materials, but does not justify autonomous deployment or direct patient self-use. Key evidence gaps remain in comprehension outcomes, workflow impact, and real-world validation.

## Introduction and background

Radiology is central to modern diagnostics, yet its outputs have traditionally remained inaccessible to patients. Reports are written in specialist terminology, and medical images are stored within picture archiving and communication systems (PACS), limiting patient understanding despite growing expectations for transparency [1-3]. Recent expansions in patient-centred care, including electronic health-record (EHR) portals and open-access initiatives, now provide patients with near-real-time access to radiology reports and, increasingly, to images [4,5]. This shift has exposed a persistent gap between information access and comprehension.

At the same time, radiologists are under increasing workload pressures. Rising imaging volumes, staffing shortages, and expanding communication expectations contribute to time constraints, cognitive load, and burnout. Report-related communication tasks such as explaining findings to clinicians, rewording reports for patients, or resolving misunderstandings are an under-recognised but significant burden [6].

**Figure 1:**
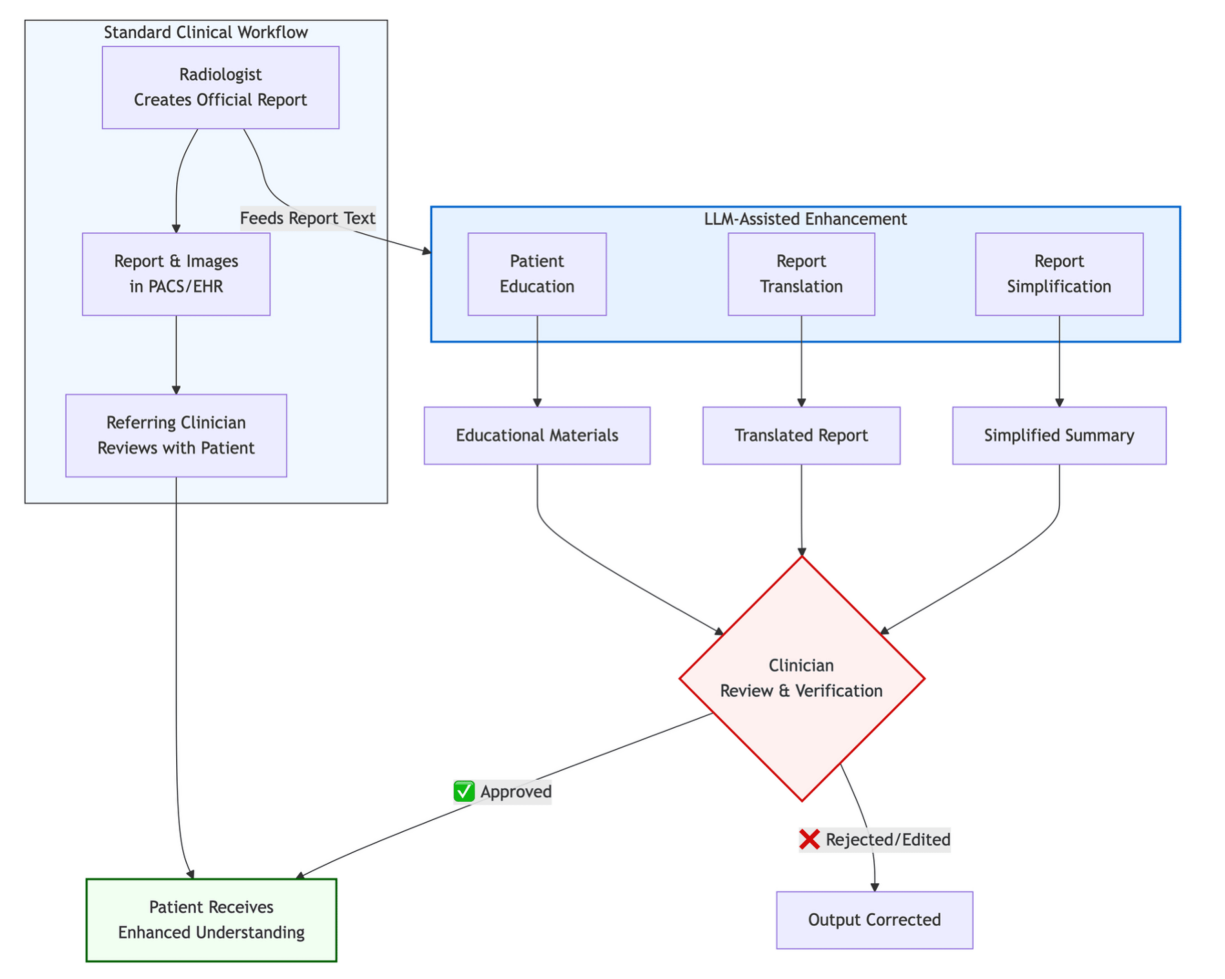
Clinician-mediated workflow for LLM-assisted radiology communication. Schematic overview of a clinician-mediated large language model (LLM) workflow for radiologist–patient communication. LLM-assisted report simplification, translation, and patient education are generated from the original radiology report and undergo mandatory clinician review and verification prior to patient release, ensuring professional oversight and accountability. Source: Author-created figure.

Across clinical medicine, artificial intelligence has been associated with improvements in diagnostic accuracy, therapeutic support, operational efficiency, and patient safety across a wide range of clinical applications [7,8]. Large language models (LLMs), including models such as GPT-4 and Gemini and their deployment within platforms such as ChatGPT and Microsoft Copilot, have demonstrated the capacity to process complex medical text and generate patient-friendly explanations [8]. Early studies show that LLMs can simplify reports [9,10], translate findings into multiple languages [11], and produce educational material; however, safe use consistently relies on clinician-mediated workflows in which outputs are reviewed, corrected, and contextualised before communication with patients (Figure 1) [12].

Within this framework, translation represents a critical and frequently encountered component of radiology communication. In multilingual healthcare systems, linguistic accessibility is a key determinant of patient understanding, particularly for individuals with limited English proficiency [13]. Translation requests are common and time-intensive for clinicians, and delays or inaccuracies may exacerbate inequities in access to imaging information. When appropriately supervised, LLM-generated translations may offer a scalable means of supporting timely, culturally sensitive communication while reducing repetitive linguistic workload [14].

Despite these potential benefits, direct patient use of LLMs remains largely unevaluated and raises concerns related to misinformation, bias, privacy, and data-protection frameworks such as the Health Insurance Portability and Accountability Act (HIPAA) and the General Data Protection Regulation (GDPR) [8,15,16]. Regulatory pathways, including the FDA’s Software as a Medical Device (SaMD) framework, the UK Medicines and Healthcare products Regulatory Agency (MHRA) AI roadmap, and the EU AI Act, currently provide limited guidance for non-diagnostic, patient-facing applications [17-19].

Clinician-mediated deployment, therefore, represents a pragmatic and safe intermediate step. It may enhance health literacy, improve patient engagement [12], reduce linguistic and educational barriers to understanding [20], and alleviate components of radiologist workload related to communication tasks, while maintaining essential professional oversight. This narrative review synthesises current evidence on clinician-mediated LLM use for radiology communication. It examines: (1) report simplification, (2) multilingual translation, (3) patient education, and (4) patient attitudes toward AI-assisted communication, alongside safety, governance, and regulatory considerations. Key terms are provided in Appendix 1.

## Review

Materials and methods

This article is a narrative review, conducted and reported in accordance with the principles of the Scale for the Assessment of Narrative Review Articles (SANRA) [21], which emphasises clarity of rationale, explicit aims, justified literature search, appropriate referencing, scientific reasoning, and meaningful presentation of evidence.

A structured but non-systematic search of PubMed, Scopus, and Google Scholar was performed for English-language literature published between January 2020 and November 2025, capturing the period in which modern large language models (LLMs) became clinically relevant. Search terms included combinations of Medical Subject Headings (MeSH): “radiology”, “medical imaging”, “large language model”, “LLM”, “ChatGPT”, “GPT-4”, “patient communication”, “report simplification”, “translation”, “patient education”, “health literacy”, “AI communication”, “clinician-mediated”. Reference lists of included studies were manually screened and back-searched to identify additional relevant publications.

Studies were included if they examined or discussed the use of large language models to simplify, translate, or explain radiology reports or imaging information for patients or lay audiences; addressed clinician-mediated or supervised workflows; reported empirical findings or provided relevant ethical, regulatory, or governance analysis; and were published in English. Studies were excluded if they focused solely on diagnostic performance without a communication component, evaluated direct unsupervised patient use (outside the scope of this review), or consisted of commentaries, case reports, or opinion pieces without substantive analysis.

Grey literature on regulation and governance was included for contextual governance analysis rather than performance evaluation. It was retrieved from professional bodies (RCR, ACR), regulatory agencies (MHRA, FDA), data-protection authorities (ICO, HHS), and international organisations (WHO).

Given the heterogeneity of study designs and evaluation metrics (readability indices, accuracy ratings, translation metrics, patient surveys), findings were synthesised narratively, consistent with SANRA expectations. Empirical studies provide the primary evidence base for performance and safety outcomes, while ethical analyses and regulatory guidance are incorporated to contextualise clinical implementation and governance requirements. These complementary sources serve different evidentiary roles and are not treated as equivalent in weight. Extracted information included: Study design and context, communication task (simplification, translation, patient education), models evaluated, imaging modalities, evaluation methods (readability, semantic fidelity, safety, patient comprehension) and governance or regulatory relevance. Themes were grouped into the key clinical domains presented in the review.

Results

A total of 49 studies examining applications of LLMs in radiology met the inclusion criteria and were synthesised narratively. Study design, patient-facing status, and primary task are systematically presented in Table 1.

**Table 1:** Summary of patient-facing large language model studies included in the review (n = 49) This table summarises the characteristics of all 49 studies included in the narrative review, classified by study design, patient-facing status, and primary task [8-12,20,22-64]. The included evidence spans prospective and retrospective evaluations, randomised controlled trials, qualitative and survey-based studies, comparative model assessments, and narrative or systematic reviews. Most studies evaluated patient-facing applications of large language models in radiology, including report simplification, readability enhancement, multilingual translation, patient education, consent support, and assessment of patient attitudes toward artificial intelligence.

Citation Number	Study	Study design	Patient-facing?	Task
[22]	Lyu et al. 2023	Prospective evaluation	Yes	Translate full radiology reports into plain language
[23]	Amin et al. 2023	Retrospective multicentre study	Yes	Simplify full radiology reports
[9]	Jeblick et al. 2024	Exploratory case study	Yes	Simplify radiology reports
[24]	Doshi et al. 2024	Quantitative analysis	Yes	Simplify “Impression” sections of reports
[10]	Rahsepar et al. 2024	Prospective comparative study	Yes	Enhance report readability
[12]	Park et al. 2024	Single-centre evaluation	Yes	Generate patient-friendly MRI summaries
[25]	Sterling et al. 2024	Quality and safety evaluation	Yes	Produce lay summaries of radiology reports
[20]	Tariq et al. 2024	Model development study (preprint)	Yes	Generate literacy-tailored summaries
[26]	Maroncelli et al. 2024	Pilot study with lay readers	Yes	Simplify breast imaging reports
[27]	Aydin et al. 2024	Scoping review	Yes	Review patient-facing LLM applications in imaging
[28]	Berigan et al. 2024	Randomised controlled trial	Yes	Provide LLM-generated summaries versus usual care
[29]	Gupta M. et al. 2024	Readability study	Yes	Simplify radiological information
[30]	Tepe et al. 2024	Quantitative evaluation	Yes	Simplify reports and classify urgency
[31]	Butler et al. 2024a	Retrospective evaluation	Yes	Simplify foot and ankle radiology reports
[32]	Butler et al. 2024b	Retrospective evaluation	Yes	Simplify knee MRI reports
[33]	Tang et al. 2024	Method development study	Yes	Generate colloquial radiology summaries
[34]	Kuckelman et al. 2024	Prospective evaluation	Yes	Summarise musculoskeletal MRI reports
[35]	Cesur et al. 2024	Quantitative evaluation	Yes	Simplify non-English MRI findings
[36]	Gupta et al. 2025	Prospective clinical study	Yes	Deliver simplified reports to oncology patients
[37]	Hu et al. 2025	Cross-model evaluation	Yes	Simplify radiology reports
[38]	Sunshine et al. 2025	Quality and understandability study	Yes	Simplify radiology report summaries
[39]	van Driel et al. 2025	Prospective evaluation	Yes	Simplify Dutch radiology reports
[40]	Stephan et al. 2025	Prospective evaluation	Yes	Simplify AI-generated dental imaging reports
[41]	Herwald et al. 2025	System development and evaluation	Yes	Provide personalised explanations and Q&A
[42]	Lee H.S. et al. 2025	Retrospective evaluation	Yes	Examine accuracy–simplification trade-offs
[43]	Bozer et al. 2025	Comparative evaluation	Yes	Generate patient-friendly imaging explanations
[44]	Butler et al. 2025	Retrospective evaluation	Yes	Simplify hand and wrist radiology reports
[45]	Can et al. 2025	Comparative model evaluation	Yes	Simplify interventional radiology reports
[46]	Sarangi et al. 2023	Simplification and translation evaluation	Yes	Simplify and translate radiology reports into lay-friendly Hindi
[47]	Meddeb et al. 2024	Multilingual translation evaluation	Yes	Translate CT and MRI free-text radiology reports across multiple languages
[48]	Gupta et al. 2024	Comparative retrospective evaluation	Yes	Translate CT report impressions into simple Hindi
[49]	Khanna et al. 2024	Pilot bilingual physician evaluation	Yes	Translate radiology reports into multiple non-English languages
[50]	Gulati et al. 2024	Evaluation/commentary with empirical testing	Yes	Translate radiology report segments into multiple languages
[11]	Terzis et al. 2025	Prospective multicentre evaluation	Yes	Near real-time translation of radiology reports into multiple languages
[8]	Keshavarz et al., 2024	Systematic review	Indirectly (patient-facing outputs assessed)	Review ChatGPT performance, pitfalls, and future perspectives in radiology
[51]	Haver et al., 2024	Retrospective exploratory evaluation	Yes	Simplify patient-centred information on breast cancer prevention and screening
[52]	Gordon et al., 2024	Prospective evaluation	Yes	Answer common imaging-related patient questions and assess readability
[53]	McCarthy et al., 2023	Comparative evaluation	Yes	Deliver interventional radiology patient education content
[54]	Zaki et al., 2024	Readability intervention evaluation	Yes	Improve readability of interventional radiology procedure descriptions
[55]	Scheschenja et al., 2024	Comparative model evaluation	Yes	Provide in-depth patient education prior to interventional radiology procedures
[56]	Hofmann & Vairavamurthy, 2024	Cross-sectional physician evaluation	Yes	Deliver interventional radiology procedural information during consent
[57]	Kaba et al., 2025	Prospective expert evaluation	Yes	Explain potential complications of interventional radiology procedures
[58]	Baghdadi et al. 2024	Cross-sectional survey study	Yes	Assess patient attitudes toward the use of AI as a diagnostic tool in radiology
[59]	Ibba et al. 2024	Cross-sectional survey study	Yes	Evaluate patient perceptions of AI–radiologist interaction
[60]	Hemphill et al. 2023	Narrative review	Yes	Synthesize patient perspectives on the implementation of AI in radiology
[61]	Royal College of Radiologists, 2025	Public perception survey / report	Yes	Assess public perceptions of AI use in radiology
[62]	Currie et al. 2024	Comparative evaluation	Yes	Generate and compare patient information in nuclear medicine using LLMs
[63]	Glenning & Gualtieri 2023	Qualitative / survey-based study	Yes	Explore patient perspectives on AI in medical imaging
[64]	Fanni & Neri 2024	Commentary with perspective synthesis	Yes	Examine patient roles and perspectives in adoption of AI in radiology

Simplification of Radiology Reports

28 studies were identified that explored LLMs as tools to generate patient-friendly summaries within supervised workflows (Table 2). These included randomised, prospective and observational evaluations, with sample sizes from 3 to 1982 radiology reports across multiple imaging modalities [8-12,20,22-64]. Across studies, LLM-generated summaries were associated with higher clarity ratings and patient-reported confidence, though the degree of revision required varied by clinical context.

**Table 2:** Studies evaluating LLM-based simplification of radiology reports for patient use Summary of published and preprint studies (2020–2025) investigating the use of LLMs to generate simplified or patient-readable versions of radiology reports [9,10,12,20,22-45]. Each study is summarised by design, cohort or dataset, imaging modality, model type, evaluation approach, and principal outcomes. “Patient-facing” denotes studies in which the primary aim was to improve accessibility, readability or comprehension of radiology outputs for patients or lay readers. Included studies encompass experimental, observational, methodological and review designs addressing patient-oriented applications of LLMs within radiology reporting workflows. This table provides illustrative examples identified through a non-systematic literature search and is not intended as an exhaustive list. Abbreviations: LLM: Large language model; RCT: Randomised controlled trial; CT: Computed tomography; MRI: Magnetic resonance imaging; MSK: Musculoskeletal; US: Ultrasound; CXR: Chest radiograph; LDCT: Low-dose computed tomography; FRES: Flesch Reading Ease Score; FKGL/FKRL: Flesch–Kincaid Grade/Reading Level; PEMAT: Patient Education Materials Assessment Tool; κ: kappa statistic.

Citation Number	Study	Study design	Patient facing?	Task	Data (n, modality)	Models	Evaluation	Key outcomes
[22]	Lyu et al. 2023	Prospective evaluation	Yes	Translate full reports into plain language	138 reports (LDCT; brain MRI)	ChatGPT-3.5; GPT-4	Radiologist accuracy & completeness ratings	Mean 4.27/5; low omissions/misinformation; GPT-4 improved results
[23]	Amin et al. 2023	Retrospective multicentre	Yes (readability-focused)	Simplify full reports	254 reports (CT/MRI/US)	ChatGPT-3.5; Bard; Bing	Readability + fidelity review	Reduced reading level; ChatGPT-3.5 & Bing most accurate
[9]	Jeblick et al. 2024	Exploratory case study	Yes	Simplify reports	3 synthetic reports	ChatGPT	Radiologist correctness/completeness	Understandable; some omissions
[24]	Doshi et al. 2024	Quantitative analysis	Yes	Simplify Impression sections	Multi-institutional set	Multiple LLMs incl. GPT-4	Expert scoring; readability	High readability; safety considerations
[10]	Rahsepar et al. 2024	Prospective comparison	Yes	Enhance readability	Varied reports	Four LLMs	Readability indices + review	Improved readability (P<.05)
[12]	Park et al. 2024	Single-centre evaluation	Yes	Patient-friendly MRI summaries	685 spine MRI reports	LLM pipeline	Quality; accuracy; consistency	Acceptable accuracy: consistency varied
[25]	Sterling et al. 2024	Quality & safety evaluation	Yes	Lay summaries	1,982 summaries (varied)	Multiple LLMs	Physician safety & quality	80.6% Very Good; modality variation
[20]	Tariq et al. 2024	Model development (preprint)	Yes	Literacy-tailored summaries	Dataset per preprint	Custom LLM	Automated + human review	Better understanding across literacy levels
[26]	Maroncelli et al. 2024	Pilot with lay readers	Yes	Simplify breast imaging reports	21 reports	ChatGPT-4o	Readability + lay comprehension	Good clarity; feasible
[27]	Aydin et al. 2024	Scoping review	Yes	Review of patient-facing uses	NA	Various LLMs	Narrative synthesis	Imaging uses emerge Nng
[28]	Berigan et al. 2024	Randomised controlled trial	Yes	Provide LLM summaries vs control	Randomised patient cohort	LLM pipeline	Patient comprehension + usability	Improved comprehension vs control
[29]	Gupta M. et al. 2024	Readability study	Yes	Simplify education material	100 texts	ChatGPT-3.5; GPT-4	Readability + expert review	Higher readability; accurate
[30]	Tepe et al. 2024	Quantitative evaluation	Yes	Simplify + classify urgency	30 reports	ChatGPT-4; Bard; Copilot	Readability; PEMAT; urgency accuracy	>70% understandability; variable urgency accuracy
[31]	Butler et al. 2024	Retrospective evaluation	Yes	Simplify foot/ankle reports	300 reports	LLM (prompted)	Readability; accuracy; hallucinations	Improved readability; accuracy ~4/5; 4–7% hallucinations
[32]	Butler et al. 2024	Retrospective evaluation	Yes	Simplify knee reports	300 reports	LLM (prompted)	Readability + hallucination rate	Improved FRES/FKGL; 2–5% hallucinations
[33]	Tang et al. 2024	Method development	Yes	Colloquial summaries	100 neuroradiology reports	General LLM + prompting	Radiologist accuracy + readability	Accuracy ↑20%; optimal 8th-grade level
[34]	Kuckelman et al. 2024	Prospective evaluation	Yes	Summaries of MSK MRI	60 MSK MRI reports	ChatGPT-4	Accuracy/completeness; kappa	Mostly correct; some harmful; k≈0.3
[35]	Cesur et al. 2024	Quantitative evaluation	Yes	Simplify Turkish MRI findings	50 synthetic findings	GPT-4; Gemini; Claude; Perplexity	Ratings + readability	GPT-4/Gemini/Claude ≈4.8–4.9/5
[36]	Gupta et al. 2025	Prospective clinical study	Yes	Deliver simplified reports to oncology patients	Oncology cohort	General LLM	Patient comprehension + satisfaction	Improved understanding & confidence
[37]	Hu et al. 2025	Cross-model evaluation	Yes	Simplify reports	Multimodality test set	Nine LLMs	Readability + quality checks	Improved readability; variable performance
[38]	Sunshine et al. 2025	Quality/understandability	Yes	Simplify report summaries	Reported in paper	General LLM	Clinician rating; error review	More accessible; oversight needed
[39]	van Driel et al. 2025	Prospective evaluation	Yes	Simplify Dutch reports	Varied reports	GPT-4	Patient comprehension + satisfaction	Significant comprehension improvements
[40]	Stephan et al. 2025	Prospective evaluation	Yes	Simplify AI-generated dental reports	Mixed modalities	ChatGPT	Patient understanding; readability	Improved communication & comprehension
[41]	Herwald et al. 2025	System development + evaluation	Yes	Personalised explanations + Q&A	Varied reports	RadGPT	Quality & educational value scoring	High-quality explanations
[42]	Lee H.S. et al. 2025	Ethics analysis	Yes	Accuracy vs simplification trade-off	Sample excerpts	GPT-4	Semantic fidelity + readability	Accuracy–clarity tension; verification needed
[43]	Bozer et al. 2025	Comparative evaluation	Yes	Patient-friendly explanations	100 CT/MRI reports	ChatGPT; Gemini; Copilot	Readability; PEMAT; expert ratings	ChatGPT best readability
[44]	Butler et al. 2025	Retrospective evaluation	Yes	Simplify hand/wrist reports	300 reports	LLM (prompted)	Readability; accuracy; hallucinations	<8th grade; hallucinations 3–6%
[45]	Can et al. 2025	Comparative model evaluation	Yes	Simplify IR reports	109 IR reports	GPT-4; GPT-3.5; Claude-3; Gemini; Mistral	Readability + qualitative scoring	GPT-4/Claude-3 best; some harmful errors

Prospective patient-facing studies, including Gupta et al. in oncology clinics and van Driel et al. in Dutch outpatients, reported higher comprehension and satisfaction, typically with only minor edits before release [36,39]. By contrast, the only randomised trial by Berigan et al. found that although summarisation improved readability, substantial manual editing was required in 80% of cases to remove speculative or overly confident statements, contributing to median delays of 2.94-4.21 days before patients received the simplified report via the portal [28].

Across large observational and safety-focused evaluations, clinician-rated accuracy for LLM-generated summaries ranged from moderate to high (≈3.9-4.3/5) [44], although inter-rater agreement was limited (κ ≈ 0.3) [34], indicating that acceptability is highly context-dependent. Clinically relevant hallucinations or qualifier distortions were uncommon but persistent, occurring in approximately 2-7% of outputs [31,32], particularly in musculoskeletal reports. Sterling et al. demonstrated feasibility at scale in 1,982 summaries, with 80.6% rated “very good” by clinicians, though error profiles varied by modality and report complexity [25].

Technical and cross-model studies by Lyu et al., Rahsepar et al., and Hu et al. showed uniformly improved readability across LLMs and modalities, with error rates of 0-5% but recurrent omission of nuance, model variability, and occasional over-simplification [10,22,37]. Across studies, readability improvements were quantitatively consistent: Flesch Reading Ease Scores (FRES) improved by 2-6 grade levels, Flesch-Kincaid Grade Level (FKGL) by 3-5 grade levels, with similar patterns in other validated indices [10,24,45]. However, Lee et al. [42] emphasise that outputs only maintain clinical accuracy at approximately an 11th-grade reading level, whereas the 7th-grade standard remains ethically preferable for informed consent and accessible communication.

Prompting strategies have also been explored. Doshi et al. found that specifying a school grade level improved readability for ChatGPT-3.5 and GPT-4 but not for Bard or Bing, showing differences in responsiveness to literacy cues between models [24]. Conversely, Amin et al. demonstrated that a minimal prompt, such as “simplify this radiology report” produced high readability and acceptable accuracy across models, showing that extensive prompt engineering may not be required for routine summarisation tasks [23].

Translation, Cultural Adaptation, and Accessibility

Six studies explored multilingual LLMs as tools to bridge language barriers in radiology by translating report content into non-English languages with sample sizes ranging from 3 to 100 radiology reports (Table 3) [11,46-50]. Across studies, performance varied substantially between languages and between high- and low-resource linguistic settings. In the largest prospective multicentre evaluation, Terzis et al. reported median expert quality scores of 4.5/5 for English, French, and Spanish outputs, compared with 4.0/5 for Russian, despite similar processing times (9-24 seconds per report), indicating that reduced accuracy was not attributable to workflow latency but to linguistic complexity [11]. Meddeb et al. [47] similarly demonstrated high translation quality for high-resource languages (English, Italian, French, German, Chinese), but observed marked reductions in medical terminology fidelity for lower-resource languages, including Swedish, Turkish, Russian, Greek, and Thai, across 100 CT and MRI free-text reports.

**Table 3:** Studies evaluating LLM-based translation of radiology reports into non-English languages Summary of published and preprint studies (2020–2025) investigating the use of LLMs for translating radiology reports into languages other than English [11, 46-50]. Each study is summarised by design, language(s) evaluated, imaging modality or report type, LLM model(s) used, evaluation framework, and principal findings. “Translation” in this context refers to the direct or simplified cross-lingual rendering of radiology report text aimed at improving accessibility for patients or clinicians with limited English proficiency. This table provides illustrative examples identified through a non-systematic literature search and is not intended as an exhaustive list. Abbreviations: CT: Computed tomography; MRI: Magnetic resonance imaging; GPT: Generative Pretrained Transformer; BLEU: Bilingual Evaluation Understudy; METEOR: Metric for Evaluation of Translation with Explicit Ordering; TER: Translation Error Rate; chrF: character-level F-score.

Citation Number	Study	Study design	Languages	Modality / Text	Models	Evaluation	Key findings
[46]	Sarangi et al. 2023	Simplification + translation evaluation	Hindi (lay-friendly Hindi)	Mixed radiology reports	ChatGPT (GPT-3.5 era)	Readability + clinician accuracy review	Generally comprehensible; some nuance lost; clinician oversight required
[47]	Meddeb et al. 2024	Multilingual translation evaluation	High-resource: English, Italian, French, German, Chinese; Low-resource: Swedish, Turkish, Russian, Greek, Thai	CT & MRI free-text reports	Multiple LLMs	Accuracy & quality across languages	High accuracy in high-resource languages; reduced fidelity in low-resource languages
[48]	Gupta et al. 2024	Comparative retrospective evaluation	Hindi (simple Hindi)	100 CT report impressions	GPT-4o; GPT-4; Gemini; Claude Opus	BLEU; METEOR; TER; chrF + expert review	Usable Hindi translations; quality varied by model and prompt
[49]	Khanna et al. 2024	Pilot bilingual physician evaluation	Vietnamese; Tagalog; Spanish; Mandarin; Arabic (+ Hindi pilot)	Selected radiology reports	ChatGPT-4	Bilingual accuracy & clarity ratings	Mixed accuracy: idiom and nuance issues noted
[50]	Gulati et al. 2024	Evaluation/commentary with empirical tests	Spanish; Arabic; Mandarin; Hindi; Vietnamese (examples shown)	Radiology report segments	ChatGPT (GPT-4 class)	Expert qualitative review	Generally accurate translations: safeguards recommended
[11]	Terzis et al. 2025	Prospective multicentre evaluation	English; French; Spanish; Russian	Mixed-modality radiology reports	GPT-4o	Translation quality + processing time	Near real-time translation feasible; lower accuracy in Russian

Targeted single-language evaluations showed comparable trends. In Hindi translations of 100 CT impression sections, Gupta et al. [48] demonstrated strong prompt dependence, with Bilingual Evaluation Understudy (BLEU) scores improving from 0.098 to 0.281 and Metric for Evaluation of Translation with Explicit Ordering (METEOR) from 0.297 to 0.547 after prompt optimisation, although clinician review still identified omission errors affecting diagnostic nuance. In contrast, Khanna et al. [49] reported mixed bilingual clinician agreement across five target languages, with lower perceived accuracy in Arabic despite acceptable performance in Hindi, Spanish, Tagalog, Vietnamese, and Mandarin. Across these studies, translations in high-resource languages received higher expert ratings than those in lower-resource or morphologically complex languages.

Patient-Education Material Generation

Eight studies evaluated LLMs as adjuncts for patient education in radiology, generating plain-language explanations, consent-style material, and responses to common imaging questions [8,51-57]. Across comparative studies, similar findings were reported: LLM outputs are generally accessible in tone and empathetic in style, yet readability frequently exceeds recommended health-literacy thresholds and content precision varies by context [51-54].

Several studies have benchmarked LLM-generated educational content against established radiology information resources. For example, McCarthy et al. compared ChatGPT-produced explanations with Society of Interventional Radiology materials and found that although the chatbot’s responses were conversational and approachable, they were more verbose, scored lower on patient-education suitability, and contained factual inaccuracies in approximately 10% of items reviewed [53]. Gordon et al. similarly demonstrated that prompting can improve accuracy and relevance, yet readability remained at a college level, well above the eighth-grade target for patient-facing information [52].

Evidence from interventional radiology (IR) patient-consent contexts showed similar results. Scheschenja et al. found that GPT-4 produced answers rated highly for accuracy and safety, with no harmful errors detected [55], while Hofmann et al. reported that practising IR clinicians judged GPT-4 explanations to be accurate and comprehensible but noted gaps in completeness and alignment with local procedural norms, particularly among senior reviewers [56]. Kaba et al. further demonstrated strong overall accuracy across 25 IR procedures, yet readability levels remained well above lay comprehension standards [57].

Overall, binary or quasi-binary measures showed non-trivial inaccuracy rates, ranging from ~11.5% incorrect answers in a societal benchmarking study (12/104) to ~13-17% inaccurate responses when answering common imaging questions (by rater-defined accuracy rubrics) [52,53]. In IR procedure education tasks rated on Likert scales, “mostly incorrect” responses occurred in ~2.3-5.3% of outputs, while no potentially harmful responses were identified in that dataset [55].

Patient Attitudes and Acceptability

Survey-based research across seven studies consistently indicates that patients are cautiously receptive to AI in radiology, particularly when it is framed as a supplement to, rather than a replacement for, clinician expertise [58-64]. Across studies, most patients continued to prefer confirmation or interpretation from a radiologist or referring clinician, even when AI-generated summaries are available [58-61].

Empirical evaluations of educational materials reinforce these themes. Currie et al. compared GPT-3.5 and GPT-4 for generating nuclear medicine patient information sheets across several procedure types, finding that GPT-4 produced outputs rated as more accurate, empathetic, and clinically appropriate, whereas GPT-3.5 content was more variable and occasionally outdated [62]. Broader qualitative analyses, such as those synthesised by Glenning et al., similarly report that patients respond positively to simplified, conversational explanations and perceive greater engagement, although a subset describe unease when the language feels excessively confident or anthropomorphic [63].

Across survey-based studies, higher trust ratings were reported among younger participants and those with greater digital literacy or familiarity with technology, while respondents across demographic groups consistently reported a preference for professional review and explicit disclosure of AI involvement [12,38,64].

Safety and Implementation

Despite encouraging early data, LLMs are not fail-safe. Their stochastic nature means that identical prompts can produce variable outputs, occasionally fabricating details or omitting key information. Robust safety processes are therefore essential. Practical implementation requirements for professionally supervised deployment are operationalised in Table 4, which provides structured governance.

**Table 4:** Safety checklist for clinician-mediated LLM use in radiology Safety checklist outlining core governance, oversight, and quality-assurance measures for the clinician-mediated use of large language models (LLMs) in radiology communication workflows. Source: Author synthesis based on [15,16]. Abbreviations: NHS DSPT: National Health Service (UK) Data Security and Protection Toolkit; GDPR: General Data Protection Regulation

Domain	Checklist Item	Purpose
Governance	Deploy within a secure, institution-approved environment	Prevents data leakage; ensures compliance with NHS DSPT and GDPR
Input control	Use anonymised, structured report text only	Eliminates inadvertent disclosure of identifiers
Clinician oversight	Mandatory review and approval before patient release	Maintains diagnostic accountability
Audit trail	Version-controlled storage of prompts and outputs	Enables traceability and quality assurance
Education	Provide training in prompt design and AI limitations	Mitigates over-reliance and misuse
Monitoring	Periodic sampling for factual accuracy and tone	Supports continuous improvement and governance reporting
Boundaries	Prohibit autonomous patient-chat functionality	Prevents unverified clinical advice or misinterpretation

Clinical Risks

General-purpose LLMs are not validated for diagnostic accuracy and may generate confident but incorrect statements [8,65-67]. Uncorrected inaccuracies could lead to inappropriate reassurance or unnecessary anxiety, for example, mishandling of qualifiers such as “cannot exclude malignancy” may invert a report’s intended message. Outputs may also diverge from clinical guidelines or fail to reflect uncertainty when summarising incidental findings [8,29,42,68]. 

Even factually correct statements can alter patient perception through changes in tone or certainty framing. Without professional mediation, such shifts risk breaching standards for communication quality and informed consent outlined in the UK GMC’s Good Medical Practice and regulatory expectations for Software as a Medical Device [17,69,70]. Clear disclaimers, transparent acknowledgement of model limitations, and escalation pathways for detected errors are therefore mandatory components of safe deployment.

Privacy and Security

Radiology data are inherently identifiable, particularly when linked to anatomy or demographics [71,72]. The use of LLMs introduces additional exposure points at data input and retention. Safe practice demands that identifiable content is never transmitted to consumer-facing platforms that store prompts for model training, an action incompatible with GDPR, HIPAA, and NHS Data Security Standards [73-75]. Risks are amplified in multimodal systems capable of reconstructing features from partially anonymised images [76].

Equity and Access

LLMs can enhance accessibility but may also widen disparities if ungoverned. Models trained predominantly on English-language, high-resource data perform less accurately and less empathetically for speakers of underrepresented languages or for patients with limited health literacy [11,49,77,78]. 

Digital inequities further influence who benefits from access to devices, reliable internet, and confidence with patient portals remains uneven [79-81]. In resource-limited contexts, dependence on free, unregulated models could amplify misinformation and erode trust [79,82]. Responsible implementation, therefore, requires multilingual validation, provision of analogue alternatives for digitally excluded patients, and ongoing audit of differential impact.

Governance and Regulation

Within supervised workflows, radiologists remain accountable for patient-facing explanations derived from LLMs. Liability rests with the clinician-endorsed output, not the underlying model. This distinction separates clinician-mediated use from unsupervised patient self-use, which falls outside the formal duty of care [83-85]. These two contexts differ materially in verification requirements, liability pathways, and documentation expectations, as summarised in Table 5.

**Table 5:** Consent, disclosure, and documentation requirements for patient-facing LLMs in radiology The table outlines recommended patient disclosures, consent mechanisms, clinical record documentation, responsible sign-off, and traceability controls to support safe, transparent, and accountable deployment. Abbreviations: AI = artificial intelligence; LLM = large language model; IR = interventional radiology; EHR = electronic health record; IRB = institutional review board; REC = research ethics committee. Source: Author synthesis. Note: Disclosure standards align with GMC Good Medical Practice [88], FDA SaMD labelling guidance [17,89], and EU MDR Annexe XIV clinical evaluation requirements [86, 90]. Research or pilot use should follow ISO 14155 and ICH-GCP for documentation, consent and audit trails [91,92].

Clinical Setting / Use Case	Minimum Disclosures to Patient	Consent Mechanism	Documentation in Clinical Record	Responsible Sign-off	Traceability / Version Control
Patient portal displaying AI-simplified report summary	AI involvement; limitations; official report prevails, when to seek care	Inline tick-box or portal disclaimer	Copy of AI summary stored with model version/date	Reporting radiologist or governance lead	Model ID, version, and prompt template retained in audit log
Translated radiology report (LLM-assisted)	Disclosure of AI translation; confirmation of bilingual review; limits of automatic translation	Portal prompt before translation	Record of review and sign-off attached to report	Bilingual clinician / radiology department	Original and translated text linked by version ID
AI-generated patient-education or consent information (e.g., IR procedures)	Statement that content was AI-generated and clinician-verified; not a substitute for face-to-face consent	Tick-box acknowledgement of AI-assisted material	Copy archived in consent record or EHR	Interventional radiologist / consent supervisor	Version control via LLM audit trail; timestamped approval
Pilot or research deployment of LLM interface	Explicit research consent; description of data handling and withdrawal rights	Written informed consent under IRB/REC approval	Full log of patient interaction stored securely	Principal investigator	Traceability per ISO 14155 / GCP standards

Current regulatory frameworks (FDA SaMD, MHRA AI programme, EU MDR/AI Act) focus on diagnostic AI and provide limited guidance for generative communication tools [77,86,87]. As a result, deployment relies on local governance rather than external certification. In practice, LLM-assisted communication should be governed similarly to PACS or EHR systems: institution-approved hosting, data protection compliance, documented verification, staff training, and audit.

For radiology departments, pragmatic governance requirements include designated clinical accountability, version control of prompts and outputs, explicit AI disclosure to patients, routine quality assurance sampling, and clear escalation pathways for detected errors or complaints. Until dedicated regulatory pathways emerge, institution-hosted, clinician-supervised deployment remains the only defensible model.

Discussion

This review synthesises current evidence on patient-facing applications of large language models (LLMs) in radiology and, to our knowledge, is the first to focus specifically on radiologist-patient communication. Across studies, the clearest conclusion is that LLMs can serve as effective communication adjuncts when embedded within clinician-mediated workflows. By contrast, direct patient self-use remains unvalidated and carries well-documented risks relating to misinformation, privacy, inequity, and lack of contextual interpretation [22,25,26,28,36,38-41,51].

A critical limitation pervades current evidence: the near-exclusive reliance on readability metrics (FRES, FKGL, Gunning Fog, SMOG, Dale-Chall, ARI) as proxies for comprehension. While 28 studies demonstrate 2-6 grade-level improvements in reading difficulty, only Berigan et al. directly tested whether patients understood content better or made different clinical decisions [28]. Readability measures linguistic surface structure, sentence length, syllable count, but not whether patients correctly interpret conditional language ("possible", "cannot exclude"), radiological uncertainty, incidental findings, or follow-up recommendations. These nuances carry significant clinical consequences: misinterpretation of qualifiers risks inappropriate reassurance or unnecessary anxiety, yet no study has evaluated whether simplified reports reduce such misunderstandings. Until comprehension is directly tested through patient interviews, decision tasks, adherence outcomes, or anxiety measures, the clinical value of improved readability remains technically demonstrated but clinically unvalidated.

Clinical Implications

Current evidence supports specific, supervised use cases in three domains: 1. Post-hoc report explanation via patient portals represents the most mature and immediately deployable application. When appended to formal reports with explicit AI disclosure and mandatory radiologist verification, LLM-generated summaries can improve patient understanding without altering diagnostic responsibility. Importantly, feasibility and turnaround time are highly sensitive to institutional verification thresholds and report complexity, underscoring that successful deployment depends more on workflow design than model selection. 2. Multilingual translation draft generation offers a pragmatic approach to addressing communication inequities for patients with limited English proficiency. Evidence supports reliable performance in high-resource languages, while translations in lower-resource or morphologically complex languages remain vulnerable to semantic drift. As a result, translation workflows must be explicitly framed as drafting tools, with bilingual clinician verification, version control, and clear escalation pathways embedded into routine practice. 3. Patient education and consent material supplementation, particularly in interventional radiology, can reduce repetitive explanatory workload by generating first-pass content. However, current outputs frequently exceed recommended health-literacy thresholds and exhibit variable factual accuracy, reinforcing that LLM-generated materials should supplement, not replace, clinician-led discussion and institutional consent processes.

Evidence Gaps and Research Priorities

Current evidence is dominated by proof-of-concept evaluations and small observational cohorts, with limited assessment of true patient comprehension or downstream behavioural impact. Key research priorities, therefore, include the development of radiology-specific comprehension metrics that extend beyond readability indices or translation scores such as BLEU; evaluation across diverse linguistic, cultural, and educational populations to avoid perpetuating inequities; and quantification of workflow, time-saving, and cost impacts, including effects on radiologist workload, burnout, and communication quality. Additional priorities include the creation of harmonised verification and transparency protocols, incorporating explicit guardrails for uncertainty, incidental findings, and follow-up recommendations, as well as the establishment of shared datasets comprising original reports, LLM-generated outputs, and clinician-verified summaries to support reproducibility and benchmarking. Multicentre collaborations will be essential to validate safe deployment at scale.

Limitations

This narrative review is not a systematic review and includes emerging and preprint literature reflective of the field's rapid development. Evidence regarding unsupervised patient use remains absent, restricting conclusions to supervised contexts. Additional limitations include potential publication bias favouring positive findings, geographic concentration of studies in high-income English-speaking countries, and heterogeneity of outcome measures precluding quantitative meta-analysis. The non-systematic search strategy, while comprehensive in scope, may have missed relevant studies in non-indexed sources or non-English publications. Grey literature on regulation and governance may not represent complete or current policy positions. As the evidence base grows, future systematic reviews and real-world evaluations will refine and extend the findings summarised here.

## Conclusions

Large language models demonstrate clear technical capability as clinician-mediated communication adjuncts in radiology, with consistent evidence of improved linguistic accessibility, functional multilingual translation in high-resource languages, and utility in generating first-pass patient education materials. However, current evidence primarily demonstrates improvements in readability rather than comprehension, leaving the clinical impact on patient understanding, decision-making, and outcomes largely unvalidated. Across use cases, safety and feasibility are determined less by model choice than by workflow design, verification thresholds, and institutional governance. Accordingly, near-term implementation should be limited to supervised applications-patient portal summaries, translation drafts with bilingual review, and supplementary educational content-delivered within secure, institution-hosted environments with explicit clinician accountability. Advancing clinical utility will require a shift toward comprehension-centred outcomes, rigorous evaluation across diverse populations, and implementation science focused on workflow, equity, and professional impact. Until such evidence matures, LLMs should be integrated cautiously to augment, not replace, radiologist expertise in patient communication.

## Appendices

Appendix 1

Glossary of Key Terms

**Table 6:** Glossary of Key Terms Glossary of key terms and abbreviations used in this review relating to large language models, clinician-mediated workflows, patient-facing radiology communication, readability and comprehension metrics, and governance concepts.

Term	Definition
Large Language Model (LLM)	A generative artificial intelligence model trained on large text corpora, capable of producing natural-language outputs used here for report simplification, translation, and patient education.
Clinician-Mediated Use	A workflow in which all AI-generated patient-facing content is reviewed, corrected, and authorised by a clinician prior to release.
Direct Patient Self-Use	Patient interaction with an LLM outside formal clinical workflows, without clinician verification or institutional governance.
Patient-Facing Content	Radiology-related information made accessible to patients, including simplified reports, translations, and educational materials.
Post-hoc Report Explanation	Simplified or plain-language summaries appended to completed radiology reports, without altering the original diagnostic content.
Plain-Language Summary	A rewritten version of a radiology report intended to improve accessibility by reducing technical vocabulary and sentence complexity.
Multilingual Translation Draft Generation	Use of LLMs to produce preliminary translations of radiology reports into non-English languages, requiring bilingual clinician verification.
Semantic Fidelity	Preservation of the original clinical meaning when simplifying or translating radiology content.
Semantic Drift	Loss or distortion of diagnostic meaning, qualifiers, or conditional phrasing despite grammatically fluent output.
Clinically Relevant Error	An inaccuracy or omission that could plausibly affect patient understanding, anxiety, decision-making, or follow-up behaviour.
Readability	Linguistic ease of text estimated using formula-based indices that assess sentence length, word complexity, and syllable count.
Comprehension	A patient’s accurate understanding of findings, uncertainty, and clinical implications, beyond surface linguistic simplicity.
Readability Metrics	Quantitative indices used to estimate textual difficulty; they do not directly measure comprehension or clinical understanding.
Flesch Reading Ease Score (FRES)	A readability metric scoring text from 0–100, calculated from average sentence length and syllables per word; higher scores indicate easier reading.
Flesch–Kincaid Grade Level (FKGL)	A readability formula estimating the U.S. school grade level required to understand a text, based on sentence length and syllable count.
Gunning Fog Index	An index estimating years of formal education needed to understand text on first reading, weighted toward sentence length and proportion of complex words.
SMOG Index	The Simple Measure of Gobbledygook; estimates years of education required based on the number of polysyllabic words in a text sample.
Dale–Chall Readability Score	A readability metric using a list of familiar words to estimate text difficulty; higher scores indicate more complex text.
Automated Readability Index (ARI)	A readability formula estimating grade level using characters per word and words per sentence rather than syllable counts.
Health-Literacy Threshold	Recommended reading level for patient-facing health information, typically between 7th and 9th grade.
Prompt Engineering	Modification of LLM input instructions to influence readability, tone, or accuracy of outputs.
Workflow-Sensitive Risk	Variation in safety, accuracy, and feasibility driven primarily by verification structure, release thresholds, and clinical context rather than model choice.
Verification Threshold	The institutional standard determining whether AI-generated content is released, revised, or rejected.
Human-in-the-Loop	Deployment model requiring active human review and approval of each AI output before clinical use.
Audit Trail	A time-stamped record of AI inputs, outputs, edits, and approvals enabling accountability and retrospective review.
Electronic Health Record (EHR) Portal	A patient-accessible digital platform providing access to radiology reports and related clinical information.
Picture Archiving and Communication System (PACS)	Secure system used to store, retrieve, and manage radiological images and reports.
Institution-Hosted Deployment	Use of LLMs within secure, healthcare-approved IT environments rather than public consumer platforms.
SANRA (Scale for the Assessment of Narrative Review Articles)	A validated framework used to guide the conduct and reporting quality of this narrative review.
